# Does Day Length Affect Winter Bird Distribution? Testing the Role of an Elusive Variable

**DOI:** 10.1371/journal.pone.0032733

**Published:** 2012-02-29

**Authors:** Luis M. Carrascal, Tomás Santos, José L. Tellería

**Affiliations:** 1 Department of Biodiversidad y Biología Evolutiva, Museo Nacional de Ciencias Naturales, MNCN-CSIC, Madrid, Spain; 2 Department of Zoología y Antropología Física, Facultad de Biología, Universidad Complutense, Madrid, Spain; University of Copenhagen, Denmark

## Abstract

Differences in day length may act as a critical factor in bird biology by introducing time constraints in energy acquisition during winter. Thus, differences in day length might operate as a main determinant of bird abundance along latitudinal gradients. This work examines the influence of day length on the abundance of wintering crested tits (*Lophophanes cristatus*) in 26 localities of Spanish juniper (*Juniperus thurifera*) dwarf woodlands (average height of 5 m) located along a latitudinal gradient in the Spanish highlands, while controlling for the influence of food availability, minimum night temperature, habitat structure and landscape characteristics. Top regression models in the AIC framework explained 56% of variance in bird numbers. All models incorporated day length as the variable with the highest magnitude effect. Food availability also played an important role, although only the crop of ripe juniper fruits, but not arthropods, positively affected crested tit abundance. Differences in vegetation structure across localities had also a strong positive effect (average tree height and juniper tree density). Geographical variation in night temperature had no influence on crested tit distribution, despite the low winter temperatures reached in these dwarf forests. This paper demonstrates for the first time that winter bird abundance increases with day length after controlling for the effect of other environmental variables. Winter average difference in day length was only 10.5 minutes per day along the 1°47′ latitudinal interval (190 km) included in this study. This amount of time, which reaches 13.5 h accumulated throughout the winter season, appears to be large enough to affect the long-term energy budget of small passerines during winter and to shape the distribution of winter bird abundance under restrictive environmental conditions.

## Introduction

Differences in day length might act as a critical influence on bird abundance during winter by introducing time constraints to energy acquisition. The reduction in day length typical of winter months should affect the time available to search for food, and should increase the time of inactivity (night) under very low temperatures. Therefore, reduction in day length should increase the foraging effort of birds to cope with high physiological requirements necessary to live under winter low temperatures. Consequently, the interacting effects of day length, environmental temperature, and food availability should strongly affect winter bird biology [1]–[10]. This is the conceptual background of many studies on wintering birds where thermoregulatory costs, mediated through food availability and ambient temperature, are at the core of many approaches to bird distribution and abundance [11]–[12].

Several approaches have supported the main effect of temperature on bird distribution in habitats located below thermo-neutral zones for the species [13]–[14], supporting the proposal of Root [15] that ceiling metabolic rates that constrain bird numbers in cold environments exist (but see [16]). Nevertheless, few approaches have explored the role of day length as an independent environmental constraint to bird abundance. Stapanian et al. [17] found a positive relationship between photoperiod and bird species richness within the same area and across different winters, but no empirical tests have been performed to identify the role of day length as an independent feature affecting bird numbers across large spatial scales. The lack of these studies may be related to the difficulties of controlling the concomitant effect of temperature and other environmental features affecting bird numbers (food availability and vegetation structure) along the latitudinal gradient which is responsible for changes in day length.

**Figure 1:**
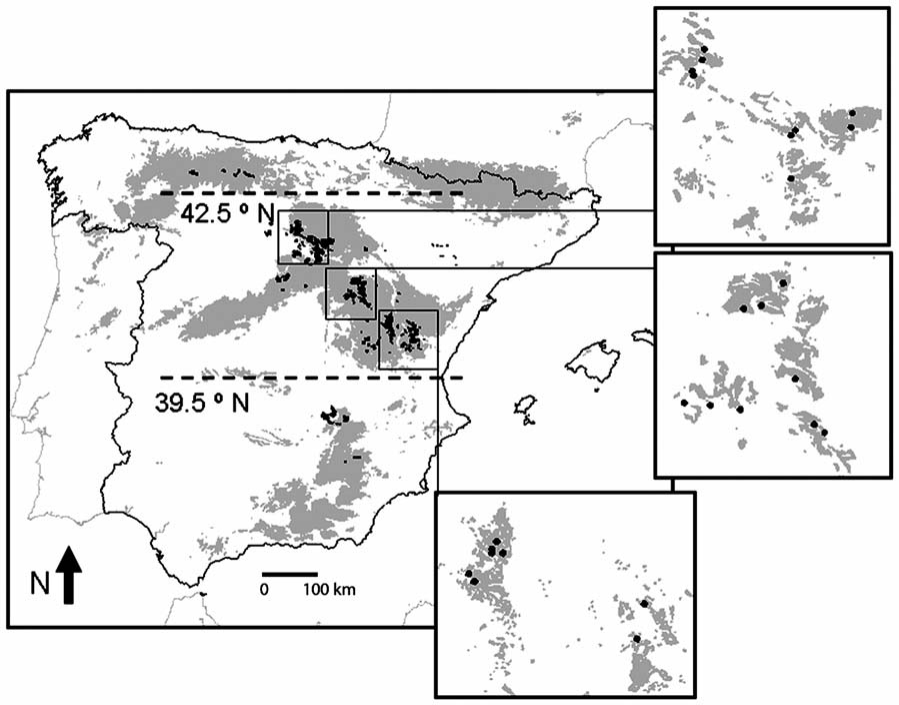
Distribution of Spanish juniper woodlands and the study localities. Grey areas depict areas over 1000 m above sea level on the Iberian Peninsula and black patches show the actual distribution of the Spanish juniper woodlands. Boxes show the spatial distribution of the study localities (black points) on the expanses of juniper woodlands (grey).

This paper tests for the first time the quantitative importance of day length, relative to other factors such as food abundance, temperature, vegetation structure and landscape characteristics, in determining the winter abundance of a small bird living under harsh environmental conditions. We carried out a large-scale study focusing on crested tit abundance (*Lophopanes cristatus*, Paridae) in Spanish juniper (*Juniperus thurifera*) dwarf woodlands distributed north to south along the cold highlands of the Iberian Peninsula (Fig. 1). Crested tit is the most abundant resident bird species in juniper woodlands both on a year-round basis and in winter, while other small birds, sensitive to energy constraints in winter, are very scarce [18]. Because of their high metabolism relative to their small body mass (11 g), crested tits spend a high percentage of the winter day searching for food [19]. We make three general predictions regarding regional variation in crested tit abundance. First, day length and air temperature, two abiotic factors related to energy constraints, will positively affect tit abundance, although the role of temperature will be of lower importance considering that birds can withstand severe cold if enough food is available (abundant fruit crops in our study system; [11]–[12]). Second, spatial variation in tit abundance will track fruit availability, especially considering the generalized low availability of winter arthropods in the temperate zone, and the fact that fruit may be critically important for winter survivorship of numerous permanent resident or short-distance migrant birds [20]. And third, crested tit abundance will increase with the development of the tree layer (in height and density), considering the marked preferences of the species for mature coniferous woodlands [21].

## Materials and Methods

### Study area and species

The study area includes the main distribution range of Spanish juniper woodlands in the Iberian Peninsula. Spanish juniper forms and dominates open woodlands, and is sometimes accompanied by isolated patches of other species (*Quercus* spp., and *Pinus nigra*; [22]). Spanish juniper is a small-sized tree that produces variable fruit crops, and the latitudinal spread of its woodlands conforms to an environmental gradient suitable to exploring the effect of day length on bird numbers while controlling for the effects of other variables such as temperature, food availability and vegetation structure. These woodlands extend over almost 125,000 hectares ([23]; Fig. 1), mostly between 800–1200 m above sea level, in areas dominated by hot summers and cold winters with annual precipitation of 400–500 mm [22]. Winter conditions are severe, with low average temperatures (mean values between 1.8°C and 4°C; the freezing period stretches from mid-October to late-May), and relatively high snowfalls (average of ten days per year; [24]). Climatological conditions during the study winters (December to 15^th^ February, 2009–2011) were characterized by frequent snowfalls (19% of the days), low minimum (−1.2°C) and average temperatures (3.2°C), and many days with minimum temperatures below 0°C (56% of the days; data from Instituto Nacional de Meteorología averaged for Burgos and Teruel meteorological stations, located at both latitudinal limits of the study region).

The study was conducted in 26 plots (0.5-km line transects, see below) arranged in three main areas encompassing the main range of Spanish juniper (Fig. 1). All studied plots were located in tracts of juniper woodlands larger than 1 km^2^. Location of study plots was chosen among the available large tracts of juniper woodlands in Spanish highlands using the GIS database Inventario Nacional Forestal II (Spanish Ministerio de Medio Ambiente), considering the maximization of the latitudinal gradient, and the accessibility by local paved roads regularly serviced by public snow ploughs. Within each locality the study plots were chosen at random, surrounded by at least 0.5 km of juniper woodlands. Researchers had no previous knowledge of temperatures, and abundances of birds, fruits and arthropods at each plot. Astronomical day length duration at winter solstice was obtained from Garmin MapSource 6 (1999–2009 Garmin Ltd), using the geographic coordinates of each study area. The latitudinal variation of the 26 study areas spanned 191 km or 1°47′02″ (between 41°59′32″ and 40°12′30″), and the difference in daylight at winter solstice between the southernmost and northernmost localities was 12 minutes (547 vs. 559 minutes). We conducted our study on public and private lands, none of which required official or legal permission by Spanish law to do observational work that did not involve the capture or manipulation of birds or junipers. In the few cases where plots were established in private woodlands, we obtained the permission of the owners after friendly visits and consultations.

Crested tit (*Lophophanes cristatus* L.; Fam. Paridae) is an almost endemic species of the western Palearctic forests, that preferentially inhabits well-developed coniferous woodlands. The greater abundances of wintering crested tits in the Iberian Peninsula are attained at mountain areas, where the species mainly finds the best habitats in terms of forest structure [25]. It is a small species (11.5–13 g) that wanders in mixed flocks with other tit species throughout the juniper woodlands, where it forages on tree branches for insects and juniper fruits (pers. obs.). Its diet includes a wide variety of animal (mainly insects and spiders) and diverse plant materials, although winter diet is dominated by seeds and fruits from diverse tree and shrub species ([21]; see [26] for Spain). Crested tit is a regular breeder and sedentary species in these juniper forests [18].

Insect availability is very restricted in the Spanish juniper woodlands during winter, while diversity of plant resources is very low and practically reduced to juniper fruits [18]. Ripe fruits are available during the autumn-winter period both on trees and on the ground, since fruits naturally fall to the ground starting in early autumn [27]. Ripe juniper fruits supply a nutritive winter food as pulp includes some lipids (3.4%) and protein (3.7%), and a high percentage of soluble carbohydrates (42.3%; [28]). Furthermore, as tits search for larvae on fruits, juniper fruits may also supply animal matter since a high percentage of fruits are parasitized by insects [29]–[30].

### Bird counts

Tits were counted along 0.5-km line transects located at the 26 study areas in winters 2009–2010 and 2010–2011 (one line transect per study area). Line transects were always directed towards the centre of the juniper woodland tracts. Line transects were surveyed on windless and rainless days, walking cross-country or along narrow dirt tracks at a low speed (1–3 km/h approximately). Counts were conducted during the first four hours after dawn and the two and a half hours before dusk on different days. Transects were surveyed both at dawn and at dusk, randomizing at which times they were surveyed. These censuses were repeated two times each winter, in December and January. The location of the line transect centre was recorded using a portable GPS receiver. We recorded all contacts heard or seen, as well as the number of individuals per contact and the perpendicular distance from the transect line. Birds detected were assigned to two different census belts according to the perpendicular distance to line transect: ≤25 m and >25 m. An index of detectability was built as the ratio between the birds observed at ≤25 m and total amount of birds observed [31]. There were no significant differences between observers in the proportion of birds observed at ≤25 m of the line transect (χ^2^ = 0.08, 2 d.f., *p* = 0.961; i.e., lack of inter-observer variation in detection probabilities). From these four counts, we recorded the mean number of crested tits detected per transect, regardless of distance to the line transect, as an index of the relative abundance in each locality.

### Habitat structure

Structure of arboreal layer was recorded in a 500×10 m belt (5,000 m^2^) along each of the 26 line transects. Tree density was estimated by counting the number of Spanish junipers and other tree species >2.5 m height; Spanish juniper was the dominant tree (95% of trees recorded), while pines (mainly *P. nigra*), holm oaks (*Quercus ilex*) and deciduous oaks (mainly *Q. faginea*) were also recorded (Table 1). Average tree height was estimated by eye, after training, for the 50 junipers nearest to each line transect. Previous training with a laser rangefinder helped to reduce inter-observer variability in distance and height estimates.

**Table 1:** Mean, standard deviation (sd) and range (min/max) of study variables in 26 juniper woodlands in Spain in winters 2009–2010 and 2010–2011.

	mean	sd	min	max	R^2^
# crested tits/500 m	1.3	1.1	0.0	4.0	0.254**
tree height (m)	5.1	0.7	3.9	6.4	0.003
# juniper trees (in 5,000 m^2^)	93.1	47.3	22.5	219.0	0.067
# oaks (in 5,000 m^2^)	1.5	4.3	0.0	17.0	0.070
# holm oaks (in 5,000 m^2^)	1.8	4.2	0.0	17.0	0.011
# pinus (in 5,000 m^2^)	1.9	6.6	0.0	29.0	0.002
fruit abundance (# fruits per tree)	357	408	10	1578	0.034
arthropod abundance (# per minute)	0.1	0.1	0.0	0.2	0.022
altitude (m)	1176	1016	9506	1314	0.236*
minimum night temperature (°C)	−1.4	0.6	−3.2	−0.4	0.054
minimum absolute temperature (°C)	−12.8	1.8	−18.3	−10.1	0.044
maximum diurnal temperature (°C)	7.6	1.2	5.3	10.1	0.003
day length at winter solstice (min)	553	4	547	559	-------

Relative abundance of tits refers to the mean number of birds counted in four censuses without detection distance limit (two censuses per winter). Values for temperatures are averages for 77 days (1 December to 15 February) in two consecutive winters. Tree height and abundances of fruits and arthropods are estimated in each juniper woodland as the average of several samples (see Methods for more details). R^2^: square of the correlation between day length at winter solstice and each variable (**: *p*<0.01; *: *p*<0.05; no correlation was significant after applying Bonferroni correction).

Several studies have shown the relative importance during winter of surrounding landscape-level variables for wildlife in relatively homogeneous habitats [32]. Thus, the landscape structure around each juniper woodland was recorded within a 1 km radius buffer around the line transect centre using GIS databases (Inventario Nacional Forestal II, 1986–1996, Spanish Ministerio de Medio Ambiente) comprised of eleven habitat categories (holm oak, Portuguese oak, pine and mixed forests, galley woodlands along rivers, shrublands, pasturelands, rocky areas, irrigated lands, cereal crops, and urban areas). A principal component analysis was carried out with the data matrix of 26 localities×11 habitat categories, extracting the first three landscape components (i.e., the 26 localities differed in one or more of these axes): PC1 – cover of agricultural landscape around each juniper woodland plot; PC2 – cover of coniferous and mixed forests dominated by *Pinus nigra*; PC3 – cover of holm oak forests).

### Fruit and arthropod abundance

Abundance of ripe fruits was assessed by using the nearest 20 Spanish junipers distributed every 25 m alongside the line transects used to count birds. We used the same junipers in both winters. Ripe juniper fruits were distinguished considering colour and size (blue or blue-black, succulent and sweet to taste; see details in [33]). We counted all ripe fruits in each juniper tree. Fruits were uniformly distributed in the juniper crown, from the lower branches to the tree top. Fruit counts were made in both winters (last week of November). Mean fruit abundance was calculated for each winter and census plot averaging fruit counts in the 20 juniper trees. Finally, we obtained the mean fruit abundance across both winters.

Arthropod availability for a forager such as the crested tit was estimated by counting all arthropods longer than 1 mm found during visual searches of 1 min length in juniper foliage and twigs (see [34] and references therein). Counts were made in the second winter 2010–2011 (14–21 January) in rainless days from 10 a.m. to 5 p.m., when temperatures reached values high enough for arthropods to be active. Twenty samples were obtained in each juniper woodland on the same juniper trees used to measure fruit availability. All prey items were identified to order and the length was approximated to the nearest millimetre *in situ* without collecting them. No arthropods were found in 93.7% of the samples. Average encounter rate with arthropods was 0.13 arthropods/min (n = 520 one-min samples). The average length of the encountered arthropods was 3.25 mm (n = 68 individuals). The main arthropod groups were Diptera which accounted for 32.4% of total individuals, larvae or pupae 25.0%, Arachnids 23.5%, Hymenoptera 7.4%, Coleoptera 7.4%, and Hemiptera 4.4%.

Because arthropod counts were not carried out exactly at the same time and under the same temperature in all localities, adjusted values were estimated considering air temperature (see below) and time of day during sampling to account for differences in these two variables among study sites. A generalized additive model was built using mean number of arthropods/minute/site as the response variable, and cubic splines of the predictor variables time of day and air temperature (n = 26). This model explained 73.8% of differences in arthropod abundance in juniper foliage, showing that differences among localities were of low magnitude (i.e., 100–73.8 = 26.2% of the observed variability).

Arthropod availability could not be estimated in all study localities in first winter 2009–2010, due to weather and logistic difficulties. Nevertheless several samples were obtained in the eight southernmost juniper woodlands (10 samples per locality in 15–17 January 2010). No arthropods were found in 97.5% of the samples, providing a similarly low arthropod abundance in juniper foliage as in the second winter (no arthropods found in 91.2% of the samples selecting the first 10 samples per locality in winter 2010–2011; *p* = 0.170 in Yates corrected Chi-square test).

### Air temperatures

Winter air temperature was assessed with one temperature logger (HOBO Pendant, Onset) placed at the centre of each line transect. Loggers were placed on thick juniper trunks covered by a dense layer of branches, oriented to the north, and at approximately 1.5 m above ground. Data loggers recorded air temperature every ten minutes from 1 December to 15 February of winters 2009–2010 and 2010–2011. For each recording day (144 measurements), average temperature, maximum daytime temperature, and minimum night temperature were obtained. Temperatures for the 77 days of the study period were averaged for each juniper woodland (see Table 1). These three temperature measurements were highly correlated across woodlands (*r*>0.8). Thus, the average minimum night temperature was selected as a measurement of the thermal state of the environment more likely to constrain bird distribution and abundance, considering both its clear functional relationship with maximum thermoregulatory costs at night, and the long duration of winter nights. We also considered average daytime temperature to be less discerning because birds may compensate thermoregulatory costs associated with daytime temperature by means of heat production from locomotor muscles during foraging activity [35]–[36]. We obtained the average minimum night temperature across both winters.

Minimum absolute temperature was below −5°C in 24.7% of the 77 days of the study period in winter 2009–2010 (range for the 26 localities: 17.1% to 30.3%) and 18.8% of days in winter 2010–2011 (range: 11.8% to 36.8%). There were three very cold periods each winter, with average minimum temperatures below −7°C for the 26 study localities; two lasted more than six consecutive days (see more details about variation among the study localities in Table 1).

### Data analyses

Relationships between the average number of tits per 0.5-km transect in each juniper woodland and the predictor variables were explored by means of linear regressions, using the information-theoretic model comparison approach. We compared alternative *a priori* models with Akaike's second-order AIC corrected for small sample sizes (AICc; [37]–[38]). Seven models were a priori defined: (1) landscape effects (the three landscape components plus altitude), (2) local habitat structure (juniper tree height and density), (3) landscape plus local habitat structure, and models testing for the effects of (4) temperature, (5) food availability and (6) day length, while controlling for habitat structure in each locality. Finally, a seventh model, considered more probable according to predictions presented in the introduction section, included day length, the most abundant food resource (fruits) and the local habitat structure variables (juniper tree height and density).

We also carried out AIC multimodel inference using all possible subsets of the predictor variables using generalized linear models (canonical distribution: gaussian; link function: identity). Only those more plausible models with ΔAICc<4 were retained for model averaging using Akaike weights. The multimodel approach should be viewed as the way to obtain model weights, not just a way to select only one model. Rather than to base inferences on a single, selected best model from an a priori set of models, inference can be based on the entire set of models using weights derived from AIC values. Akaike weights are summed for all models containing predictor variable x_j_, for *j* = 1, …, R models (denoted by w_+_(*j*)). The predictor variable with the largest predictor weight, w_+_(*j*), is estimated to be the most important, while the variable with the smallest sum is estimated to be the least important predictor. Then, by using the weighted average for that parameter across models (e.g., standardized regression coefficient, β*_p_*, for p predictor variables), inference is based on the entire set of models; for some models, β*_p_* = 0, if x*_p_* is not in those models. This approach has both practical and philosophical advantages, as it is based on the Kullback-Leibler information theory. A model-averaged estimator has a more honest measure of precision and reduced bias compared to the estimator from just the selected best model [38]. Standardized regression coefficients (β) were obtained in regression analyses (i.e., analyses are carried out with standardized variables, so that their averages are zero and variances are 1).

The influence of spatial location and proximity of the 26 juniper woodlands on the observed patterns of variation in crested tit abundance was tested by means of a two-order polynomial of latitude and longitude, thus performing a trend surface analysis [39]. The residuals of the regression model including those predictor variables selected by the information–theoretic approach did not show a clear spatial autocorrelation pattern (18.1% of variation in the residuals was explained by a two-order polynomial of latitude and longitude, *p* = 0.873).

All statistical analyses were conducted in Statistica 9 (StatSoft Inc, Tulsa, Oklahoma).

## Results

Bird numbers, woodland structure, altitude, and climatic features showed much variation among localities (Table 1). Day length alone accounted for 25% of the observed spatial variation in crested tit abundance across study areas (see R^2^ in Table 1). Day length was positively correlated with altitude (*r* = 0.49), both variables sharing approximately one-fourth of their variance. The remaining relationships between day length and the explanatory variables were all nonsignificant (*p*>0.15) and accounted for less than 10% of variance.

From the seven *a priori* models considered, that including day length, fruit abundance and local habitat structure variables (juniper height and density) was the one with the highest strength of evidence (according to the lowest AICc value), and explained the largest amount of variance (R^2^ = 57%). The four predictor variables had a positive influence on crested tit abundance. The magnitude effect, measured by the standardized regression coefficient (β), was highest for day length (β = 0.55, se = 0.15), followed by juniper tree height (β = 0.42, se = 0.16), fruit abundance (β = 0.29, se = 0.15) and juniper tree density (β = 0.28, se = 0.16). The model including landscape and habitat structure variables had the lowest strength of evidence.

**Figure 2:**
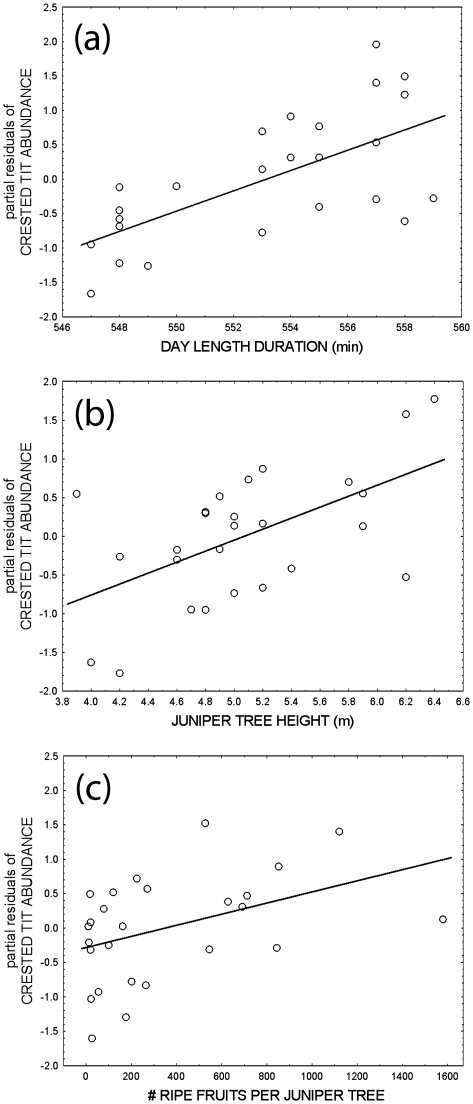
Partial residual plots illustrating the influence of day length at winter solstice (a), average height of juniper trees (b) and availability of ripe juniper fruits (c) on the relative abundance of crested tits in Spanish juniper woodlands during winter. N = 26 woodlands.

The multimodel inference approach, considering different combinations of predictors (including habitat structure variables, minimum temperature, arthropod and fruit availability and day length) selected eight models with ΔAICc<4 to explain the variation in mean number of tits per transect in the 26 studied juniper woodlands. The percentage of explained variance in bird numbers was 56% (weighted average of R^2^ for the eight selected models using model weights W_i_). The Akaike multimodel inference supported the prominent role of day length, habitat structure, and fruit availability on bird numbers (Table 2). All regression models with ΔAICc<4 incorporated day length at winter solstice (sum of Akaike weights, ΣW_i_ = 1.00), which was also the variable with the highest magnitude effect (weighted average of beta regression coefficient using Akaike weights = 0.55; see Fig. 2 for the partial residual plot of day length with the relative abundance of crested tits after controlling for all other variables). Average tree height also had maximum strength of evidence according to the sum of Akaike weights (ΣW_i_ = 1.00), although its magnitude effect was lower than that observed for day length (weighted average of beta = 0.43; see partial residual plot in Fig. 2). Other important variables were density of juniper trees and fruit abundance (ΣW_i_>0.90), although their magnitude effects, measured by β regression coefficients, were low (0.27; see partial residual plot in Fig. 2). The remaining predictor variables had very low strength of evidence and magnitude effects (sum of Akaike weights <0.26 and absolute values of weighted β<0.02; Table 2). The above mentioned effects of predictor variables on crested tit winter abundance in juniper woodlands did not change between the two study years (*p*>0.1 in the parallelism tests carried out with repeated measures ANCOVA using those predictor variables with ΣW_i_>0.90 –day length, fruit abundance, tree height and tree density–).

**Table 2:** Alternative models for the relative abundance of crested tits in juniper woodlands of Spain during winter, and results of multimodel inference using all predictor variables.

	AICc	ΔAICc	K	Wi	DL	FR	AR	Tm	hJ	#J	L1	L2	L3	ALT
**A priori models**														
HB+Fruits+DL	67.8	0.0	5	0.615	X	X			X	X				
HB+Day length (DL)	68.8	1.0	4	0.379	X				X	X				
Habitat (HB)	78.8	10.9	3	0.003					X	X				
HB+Food	80.1	12.3	5	0.001		X	X		X	X				
Landscape	80.4	12.6	5	0.001							X	X	X	X
HB+Temperature	81.9	14.1	4	0.001				X	X	X				
HB+Landscape	85.9	18.1	7	0.000					X	X	X	X	X	X
**Multimodel inference (8 models with** Δ**AICc<4)**														
ΣW_i_					1.00	0.92	0.24	0.10	1.00	0.94	0.26	0.09	0.00	0.00
weighted average					0.55	0.27	0.00	0.01	0.43	0.27	−0.02	0.01	0.00	0.00

Sample size is 26 juniper woodlands. X: variables included in the *a priori* models. Multimodel inference averages the first eight models with ΔAICc<4 using model weights (W_i_). Values presented for variables in multimodel inference are weighted averages of standardized beta regression coefficients obtained in generalized linear models considering model weights W_i_ (beta values inform about the magnitude and sign of the partial relationships of the predictor variables with the relative abundance of crested tits). AICc: AIC corrected for small sample sizes. ΣW_i_: sum of Akaike weights for each variable considering those models where they were selected in AIC multimodel inference. K: number of predictor variables+intercept. Habitat variables (HB): hJ - average height of juniper trees; #J - number of juniper trees per 5,000 m^2^. Tm: minimum night temperature. Food variables: AR - arthropod abundance; FR - fruit abundance index. DL: day length at winter solstice; Landscape variables: ALT - altitude; L1 - multivariate gradient of increasing cover of agricultural landscape around each juniper woodland plot; L2 - multivariate gradient of increasing cover of coniferous and mixed forests dominated by *Pinus nigra*; L3 - multivariate gradient of increasing cover of holm oak, *Quercus ilex*, forests.

In summary, the winter relative abundance of crested tits in juniper woodlands increased with day length, density and development of juniper tree woodlands, and availability of ripe juniper fruits; this pattern of covariation between bird numbers and environmental factors remained stable across the two study years.

## Discussion

Day length may act as a critical factor in winter bird biology by introducing time constraints in energy acquisition during winter. Thus, differences in day length might operate as a main determinant of bird abundance along latitudinal gradients. Day length was the main explanatory variable on the abundance of crested tits occurring in Spanish juniper woodlands of the Iberian highlands, in spite of its narrow variation across localities compared to vegetation structure variables, temperature or food availability (Table 1). Moreover, daytime duration alone accounted for 25.4% of the total explained variance in tit abundance (model average of R^2^ = 56.3% using Akaike weights). The importance of day length is more striking as this variable is weakly related to vegetation structure and temperature in the sample of 26 study areas (see Table 1). Therefore, even within a relatively small geographical range spanning 1°47′ of latitude, the subtle latitudinal variation in day length was of paramount importance, and was of a higher magnitude than other variables, in determining winter bird abundance.

The maximum day length difference between the two extreme localities in the latitudinal gradient was as low as 12 minutes in winter solstice. It would seem that such a difference is not great enough to influence bird physiology, and thus translate into population implications. Nevertheless, this small difference becomes energetically relevant when considered throughout the winter. Considering our study time span (1^th^ December to 15^th^ February), the average difference in day length between the northern- and southern-most localities is 10.5 minutes per day. This average difference accumulated over 77 winter days amounts to 13.5 h of daytime that is not available for foraging and is added to the time spent resting at night time. The strong evidence of our results suggests that the accumulated difference in daytime length is enough to have population implications on large spatial scales over the long-term energy budget of this small passerine [14], [40].

Our results also support the predictable influence of vegetation structure, with juniper tree height and density positively affecting crested tit numbers. Several studies have supported the importance of habitat structure in winter bird abundance (e.g., [41] for the Mediterranean region), although others have shown that vegetation structure played only a minor role in predicting bird distribution and density [42]. Variations in tree height and density are expected to play an important role in these dwarf woodlands, as crested tit inhabits well-developed coniferous forests in the south western Palearctic [21] (see [26] for the Iberian peninsula), and feed preferentially in the highest tree layers of coniferous trees even in winter ([43], [44] for Spain).

The important role of food abundance on the large-scale pattern of crested tit abundance is consistent with the fact that winter survival depends primarily on obtaining enough food for self-maintenance in resident populations of small passerines in cold areas [14], [45]. The importance of fruit availability in determining abundance of consumers is also reinforced by the high removal rate of fruits by birds under cold winter conditions [20], which are characteristic of the continental climate where Spanish junipers grow. Experimentally-manipulated food availability by large-scale diffuse feeding of populations has demonstrated the importance of food resources on winter population abundance of small birds or increased reproductive success in the subsequent breeding season [46]–[47]. Greater abundance of food resources will increase habitat suitability by reducing the amount of time required for foraging during the few light hours of winter days. Nevertheless, of the two food resources considered in this study, only availability of ripe juniper fruits exerted an important influence on bird abundance (compare Akaike weights and β coefficients of fruit and arthropod availability in Table 2). The striking contrast between the influence of these two food resources can be parsimoniously explained by considering the overwhelming abundance of fruits compared to arthropods (several orders of magnitude according to biomass). Arthropod abundance is likely limited by very low diurnal temperatures in the highlands occupied by juniper woodlands that constrain the activity of poikilotherm insects and arachnids. This result agrees with previous studies demonstrating the primacy of food abundance in determining occupancy and density of fruit consumers inhabiting woodlands during the non-breeding season [42], [48]–[49].

Higher temperatures may reduce energy demands [50] and are likely to improve the quality of the winter foraging environment [51]. Low temperatures and long winter nights are associated with an increased risk of starvation through body reserves regulation [4], [52], [53]. Temperatures in our study area were considerably lower than the lower critical temperature (usually below 20°C for many winter acclimated species in temperate areas; [50]), ranging from 5.3 to 10.1°C for average maximum diurnal temperature, and −3.2 to −0.4°C for average minimum nocturnal temperature. Therefore, local distribution of resident birds in winter should match the spatial variation of temperature. Nevertheless, there was no correlation between temperature and crested tit abundance. Our results suggest that birds can probably withstand severe cold if enough food is available (abundant fruit crops in our study system) and there is enough day light to forage in.

In conclusion, this paper demonstrates for the first time that daytime length has great importance in determining winter bird distribution, regardless of the effects of well-known biotic factors such as food availability and vegetation structure. It highlights the importance of more time available to foraging during the short cold winter days, even in large-scale distribution patterns.
